# Supplementary material for: DMRforPairs: identifying Differentially Methylated Regions between unique samples using array based methylation profiles
Source: BMC Bioinformatics. 2014 May 15;15:141. doi: 10.1186/1471-2105-15-141 (PMC4046028; doi:10.1186/1471-2105-15-141)
Supplement: Additional file 2 — DMRforPairs output for the comparison of A431-MCF7 and NA17018-NA17105. Please start from the HTML files in each folder. Available via the BMC Bioinformatics website. [file 1471-2105-15-141-S2.zip › 1394847754114233_MOESM2_ESM/A431_MCF7/figures/22890.pdf]

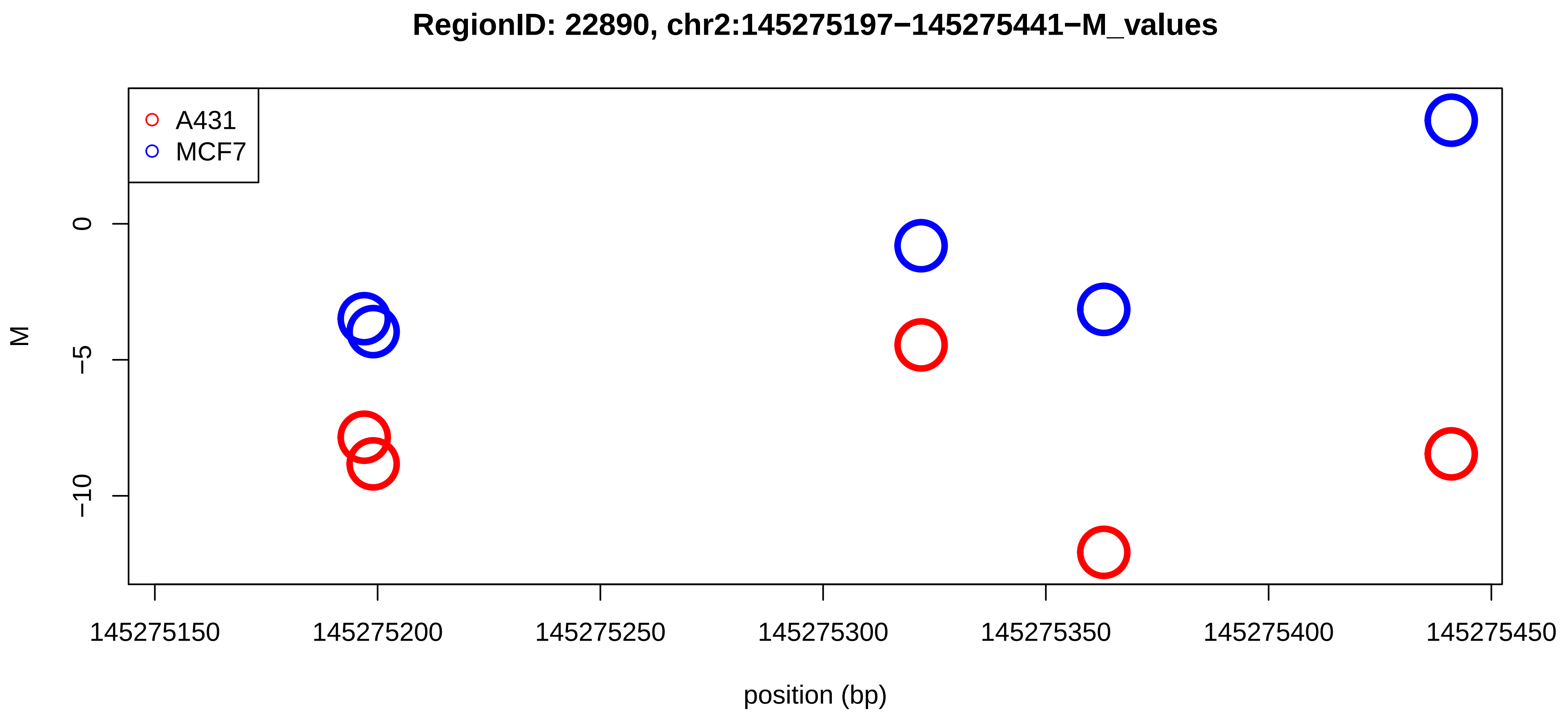
RegionID: 22890, chr2:145275197−145275441−M_values
A431
MCF7
M
0
−5
−10
145275150
145275200
145275250
145275300
145275350
145275400
145275450
position (bp)

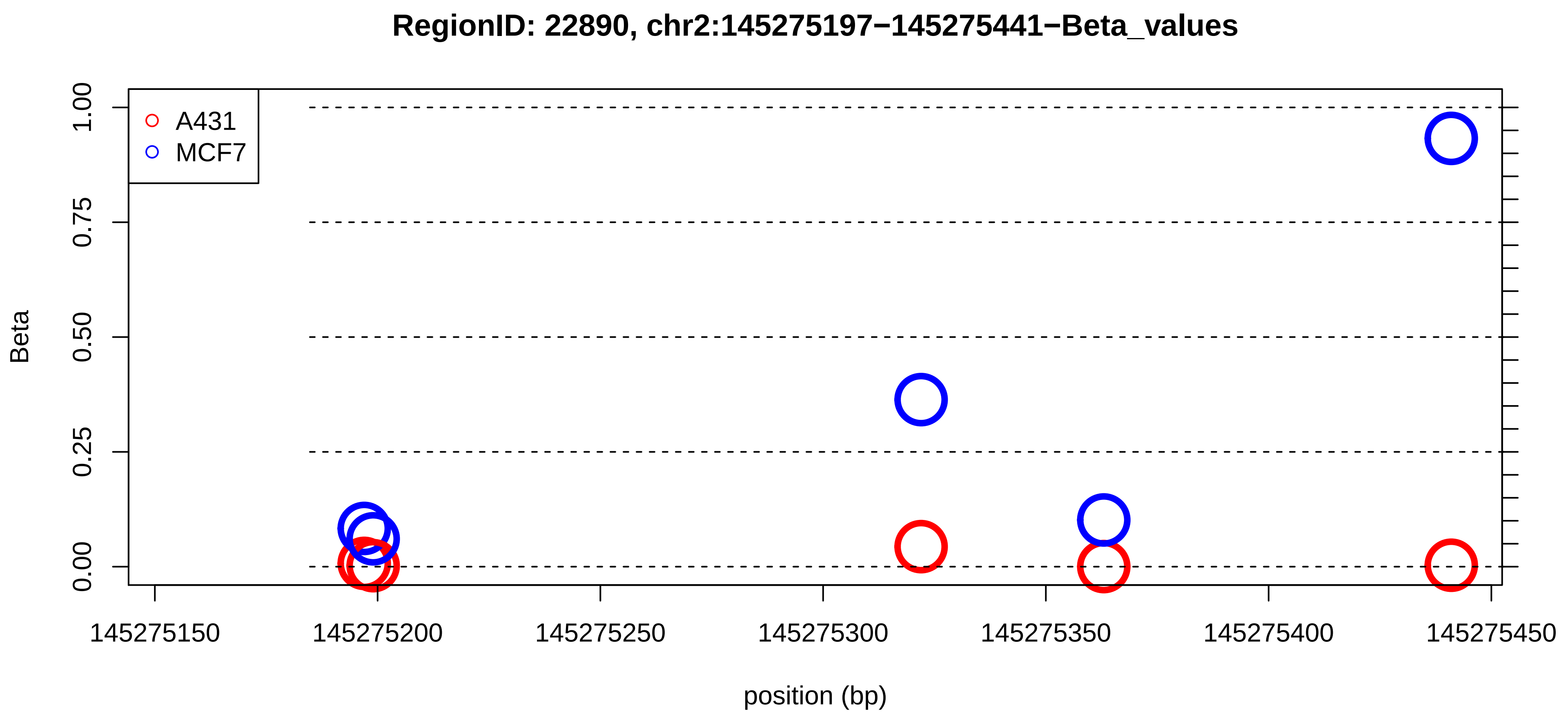
RegionID: 22890, chr2:145275197−145275441−Beta_values
A431
MCF7
Beta
1.00
0.75
0.50
0.25
0.00
145275150
145275200
145275250
145275300
145275350
145275400
145275450
position (bp)

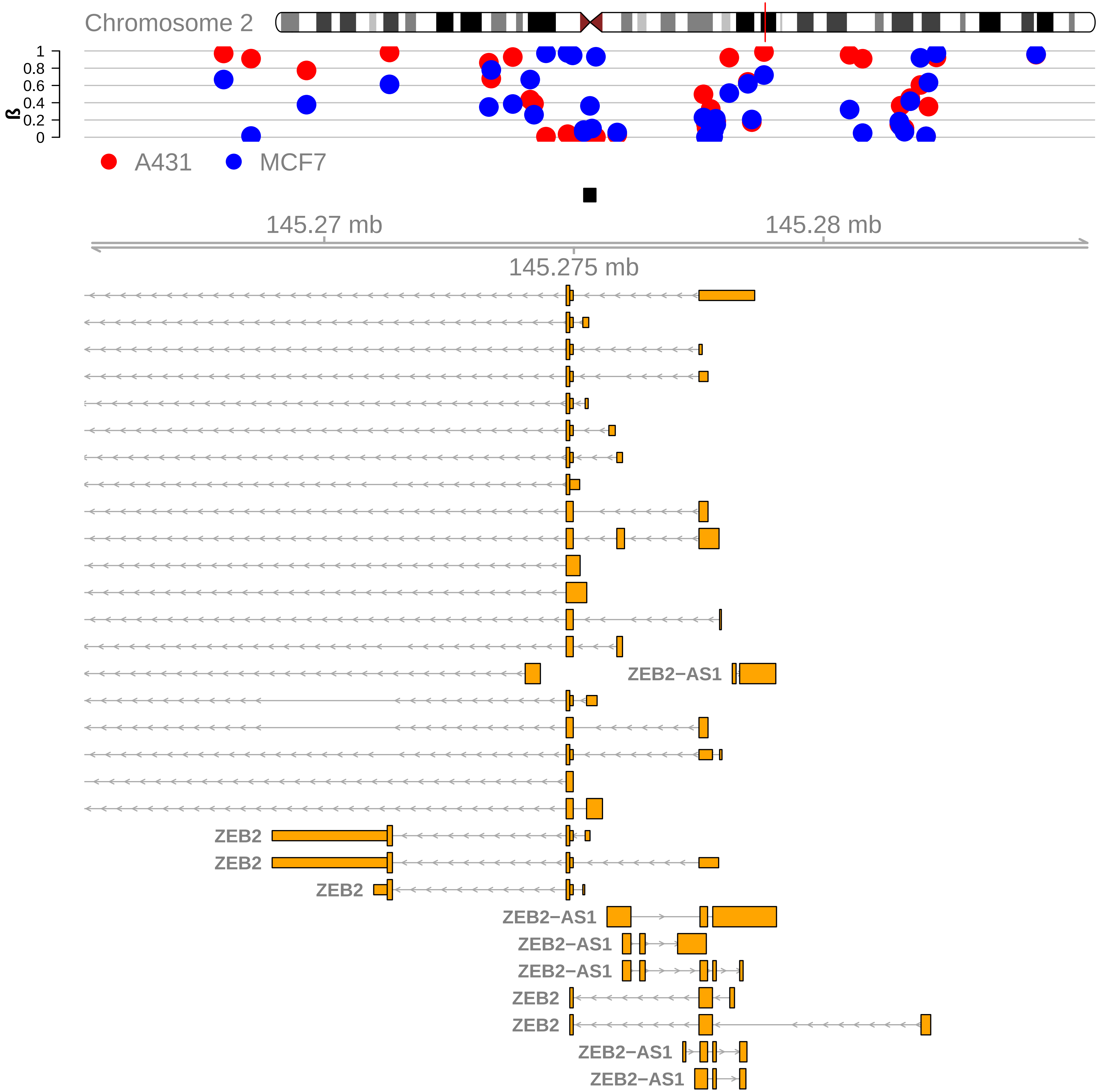
Chromosome 2
ß
1
0.8
0.6
0.4
0.2
0
A431
MCF7
145.27 mb
145.28 mb
145.275 mb
ZEB2−AS1
ZEB2
ZEB2
ZEB2
ZEB2−AS1
ZEB2−AS1
ZEB2−AS1
ZEB2
ZEB2
ZEB2−AS1
ZEB2−AS1
